# Molecular choreography of E1 enzymes in ubiquitin-like protein cascades: New insights into dynamics and specificity

**DOI:** 10.1016/j.jbc.2025.110415

**Published:** 2025-06-24

**Authors:** Caleb M. Stratton, Pirouz Ebadi, Shaun K. Olsen

**Affiliations:** Department of Biochemistry & Structural Biology and Greehey Children's Cancer Research Institute, The University of Texas Health Science Center at San Antonio, San Antonio, Texas, USA

**Keywords:** E1, E2, adenylation, thioester, transthioesterification, ubiquitin, ubiquitin-like protein, SUMO, NEDD8, structure-function, conformational change, enzyme mechanism, enzyme structure

## Abstract

In 2004, Aaron Ciechanover, Avram Hershko, and Irwin Rose were awarded the Nobel Prize in Chemistry for their groundbreaking work uncovering the stepwise, ATP-dependent degradation of cellular proteins. These studies laid the foundation for understanding ubiquitin and ubiquitin-like proteins (Ubls), an evolutionary conserved family of modifiers that mediate diverse cellular processes. The ubiquitin/Ubl system operates through a reaction cascade involving E1 activating, E2 conjugating, and E3 ligating enzymes. As the initiating enzymes, E1s catalyze Ubl adenylation, thiolation, and thioester transfer to their cognate E2s. Despite their conserved architecture, E1s exhibit strict specificity for different Ubls and E2s, a critical feature for maintaining cellular homeostasis. While the molecular mechanisms underlying E1 interactions and activities remain incompletely understood, structural studies have provided key insights into the dynamic changes that accompany Ubl activation and transfer. This review highlights recent structures that build upon foundational biochemical research, elucidating the determinants of activity, specificity, and novel regulatory mechanisms governing E1 enzymes. We examine how conformational changes drive the transition from an adenylate-competent to a thioester-competent state and how these rearrangements facilitate interactions with Ubls and E2s while advancing the reaction cycle. Additionally, we explore recent insights into a prokaryotic E1-E2–like fusion that is structurally homologous to the noncanonical eukaryotic E1 ATG7, revealing its role in activating and conjugating a non-Ubl substrate and its implications for the evolutionarily trajectory of Ubl cascades. Finally, we discuss the current landscape of E1 inhibitors under investigation as potential anticancer therapies, as well as prospects for future investigations.

Modification of substrates by the 76-residue protein ubiquitin (Ub) and ubiquitin-like proteins (Ubls) regulates essential eukaryotic processes, including protein degradation, cell division, autophagy, and immunity (Fig. 1) (1, 2). These proteins share a highly conserved tertiary structure, characterized by a central α-helix embraced by five β-strands, forming the distinctive β-grasp domain (3, 4, 5). This structural conservation supports diverse but specific interactions with downstream enzymes and substrates.

**Figure 1:**
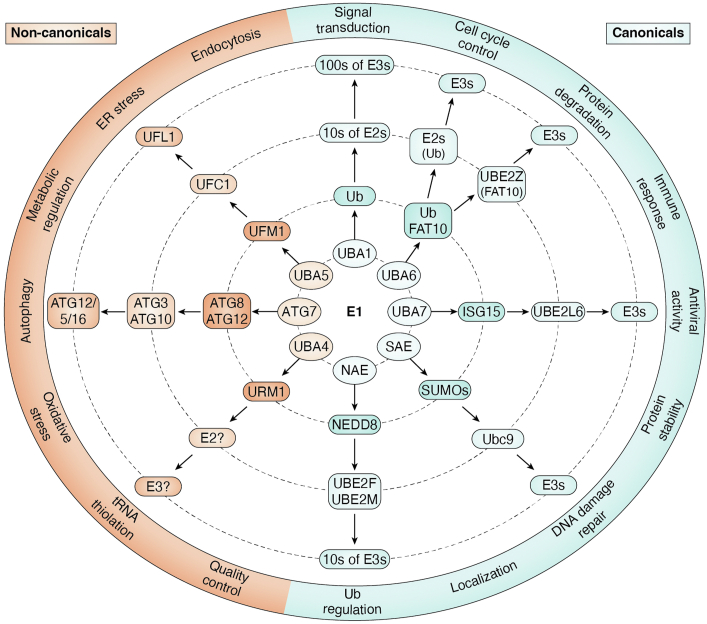
**The E1 locus of control.** E1 enzymes activate specific Ubls and catalyze their transfer to cognate E2s. These E2s interact with a distinct set of E3 ligases that in turn provide the substrate specificity needed to regulate a variety of cellular functions. The concentric layers of the diagram represent this hierarchical cascade: the inner ovals depict E1 enzymes, followed by *darker shapes* representing their cognate Ubls. The next ring contains E2 enzymes, and the subsequent ring shows associated E3 ligases. The outermost layer represents cellular functions influenced by these pathways, with approximate positioning reflecting functional roles. The absence of strict boundaries in the outer shell reflects the interconnected and overlapping nature of these processes. Ubl, ubiquitin-like protein.

Ligation of a Ubl (or Ubls) to a substrate requires the coordinated action of three protein classes: activating E1 enzymes, conjugating E2 enzymes, and ligating E3 enzymes (6, 7, 8). Activation of a Ubl by an E1 and its transfer to an E2 is a multistep, ATP-driven process involving specialized domains that ensure specificity for the E1’s cognate Ubl (or Ubls) and the potentially numerous E2s it can conjugate to (Fig. 1) (9, 10, 11). The human genome encodes eight distinct E1 enzymes, each with unique domain features that shape the architecture of a highly differentiated enzymatic cascade. This cascade governs nearly every aspect of cellular homeostasis, and its dysregulation has been implicated in autoimmune disorders, cancers, and neurodegenerative diseases (12, 13, 14, 15). Consequently, the enzymes responsible for Ubl-modification have emerged as attractive targets for drug development and therapeutic intervention (16).

Recent advancements in structural biology have deepened our understanding of Ubl activation *via* activating E1 enzymes and their subsequent conjugation to E2s. These studies have uncovered critical interactions for recognizing cognate E2s and shed light on the conformational changes involved in Ubl adenylation, thioester bond formation with E1, E2 binding and orientation, and the transthioesterification handoff of the Ubl to the E2. This review examines these findings in detail, highlighting both the similarities and how these mechanisms vary across the eight classes of E1s.

## Canonical E1s

### Ubiquitin E1

Ubiquitin E1–activating enzyme (UBA1) is ubiquitously expressed and essential for viability. Complete knockout of UBA1 in mice causes early embryonic lethality (17), a phenotype also observed in other species. For example, a temperature-sensitive *uba1* allele in *Caenorhabditis elegans* leads to embryonic and larval lethality, as well as male-specific paralysis and tail defects (18). In cultured cells, loss-of-function mutations impair growth and cause cell cycle arrest (19, 20, 21, 22, 23). Conditional depletion of UBA1 in mice produces tissue-specific phenotypes, including lymphoid T and B cell death, reduced platelet production from megakaryocytes, and autoinflammatory dermatitis and hair loss when UBA1 is deleted in neutrophils (24). Notably, the neutrophil-specific KO model closely resembles VEXAS syndrome, a late-onset autoinflammatory disorder caused by somatic UBA1 mutations in hematopoietic stem cells that primarily affects men (25, 26, 27).

UBA1 has also been implicated in diseases of the nervous system. A rare hemizygous missense mutation that impairs UBA1 function causes X-linked infantile spinal muscular atrophy (28, 29, 30), and reduced UBA1 expression has been reported in Huntington’s disease (31, 32). No null or truncating UBA1 mutations are known to cause disease in humans, likely because such mutations result in embryonic lethality before clinical symptoms could manifest (33). As the gatekeeper of the Ub cascade, UBA1 is a master regulator of protein homeostasis, and its dysfunction contributes to a wide range of developmental, neurodegenerative, inflammatory, and neoplastic disorders.

Ub, along with its activating, conjugating and ligating machinery, represents one of the most extensively studied systems in eukaryotic cells, largely due to its central role in proteasome activity and cellular regulation (34, 35). The breadth of Ub’s influence stems from its compact structure and its ability to mediate specific protein–protein interactions (36). Substrates can be ubiquitinated at key lysines, affecting their cellular localization or fate (37), and in other cases, fate is determined from multiple Ub modifications on the same substrate (38). Crucially, Ub itself can be further ubiquitinated—either at its N terminus or at any of its seven lysine residues—forming linear or branched chains that encode distinct cellular signals. For example, K48-linked chains typically mark substrates for proteasomal degradation (39), while K48/K63-branched chains can modulate signaling cascades (40).

Additional layers of regulation arise from the specific site of Ub attachment on the substrate coupled with the topology of the Ub chain (41, 42, 43). To achieve such precise control, UBA1 must maintain strict specificity for Ub and coordinate interactions with tens of E2 enzymes, which in turn can interact with hundreds of E3s (44). Given the wealth of structural and biochemical data available on this system, and the structural similarities among canonical E1s, UBA1 will serve as the benchmark for understanding the mechanisms of other E1 enzymes.

#### Domain structure of Uba1

**Figure 2:**
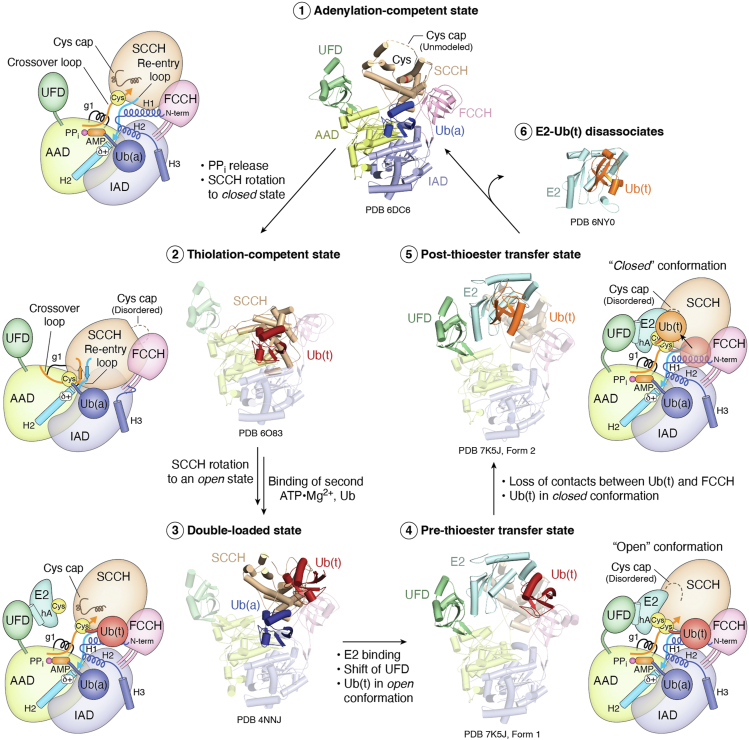
**Conformational changes during the E2 catalytic cycle.***1*, binding of ATP-Mg^2+^ and Ub. *2*, release of PP_i_ triggers a rotation of the SCCH domain to a “closed” conformation, repositioning the catalytic cysteine and remodeling the adenylation site into a thioester-competent form. *3*, following formation of the thioester bond and release of AMP, the SCCH returns to the open conformation, allowing for another ATP-Mg^2+^ and Ub to bind. *4*, E2 binds to the UFD, which shifts toward the SCCH domain to bring the catalytic cysteines of the E2 and SCCH into proximity. *5*, contacts between Ub(t) and the FCCH domain are lost as contacts are formed between Ub(t) and E2, facilitating disassociation. *6*, E2-Ub(t) disassociates to interact with E3 ligases. Ub(a) is the noncovalently bound Ub-adenylate, while Ub(t) is the thioester-linked Ub. FCCH, first catalytic cysteine half-domain; PPi, pyrophosphate; SCCH, second catalytic cysteine half-domain; Ub, ubiquitin; UFD, Ub-fold domain.

UBA1 is a large 118 kDa multidomain enzyme, with each domain playing a distinct mechanistic role in catalyzing adenylation and thioester bond formation (Fig. 2). The core of UBA1 consists of two rigid, pseudosymmetric adenylation domains (ADs): one active (active adenylation domain, AAD) and one inactive (inactive adenylation domain, IAD) (11, 45). The AAD catalyzes the adenylation reaction by binding Mg^2+^, ATP, and Ub. Both domains contain insertions of catalytic cysteine half domains. The IAD includes the first catalytic cysteine half-domain (FCCH), which is tethered to the IAD by the β7 and β14 loops. Following the FCCH, an additional insertion into the IAD forms a four-helix bundle (4HB) that appears to block Ub binding to the IAD.

The second catalytic cysteine half-domain (SCCH) is inserted into the AAD through two loops: the crossover loop, so named as it crosses near/over the protein’s C terminus, and the re-entry loop, which returns from the SCCH to form a helix within the AAD. The SCCH also harbors the catalytic cysteine that forms a thioester bond with the C terminus of Ub, protected prior to thioester bond formation by a flexible loop region known as the cys-cap. The C terminus of UBA1 includes a Ub-fold domain (UFD) and is tethered to the AAD by a single loop.

When viewed from the “front” of the enzyme (where Ub binds for adenylation), the overall structure of UBA1 resembles a left hand: the UFD forms the thumb, the SCCH represents the fingers, the FCCH corresponds to the pinky, and the AAD and IAD together make up the palm. All canonical E1 enzymes share a similar overall architecture and catalytic mechanism, though notable differences exist in domain composition—for example, the E1s for SUMO and NEDD8 function as heterodimers and vary significantly at their respective cysteine half domains.

Importantly, UBA1 adopts several distinct conformational states (46, 47, 48). The SCCH domain transitions between an “open” conformation, where the catalytic cysteine is greater than 35 Å away from the adenylation active site, and a “closed” conformation, where the catalytic cysteine is positioned within the restructured adenylation active site. Similarly, the UFD alternates between a “distal” conformation, positioned away from the SCCH domain, and the “proximal” conformation, where it is positioned closer to the SCCH domain. As will be discussed below, these distinct conformational states of the UFD and SCCH domains are important mechanistic features for the adenylation and thioester bond formation steps catalyzed by canonical E1s.

#### Mechanism of Ub activation

Elegant enzyme kinetics studies from the 1980s (Haas and Rose, JBC 1982; Goldberg, JCB 1983) papers revealed a type of ATP-dependent protein degradation distinct from the well-established, ATP-independent mechanism seen with lysozyme (9, 49). These studies would reveal the Ub proteasome system, with subsequent studies revealing that activation of Ub follows a pseudo-ordered substrate binding mechanism, where ATP-Mg^2+^ preferentially binds first, followed by Ub (9, 10, 50). ATP-Mg^2+^ binding is stabilized by several residues, including a highly conserved aspartate (Asp576 in hUBA1), which coordinates the Mg^2+^ ion interacting with the charged oxygens of the α- and β-phosphates. Kinetic analysis of thioester formation, combined with site-directed mutagenesis of Asp576, has demonstrated this preferential binding of ATP-Mg^2+^ (50). Substituting Asp576 with alanine reduces the E1 enzyme’s affinity for ATP-Mg^2+^, disrupts Ub binding, and destabilizes the Ub-adenylate transition state. Similar results have been observed on mutation of other residues shown to be involved in ATP-Mg^2+^ binding (50). These findings have been recapitulated in related E1 enzymes and in the ancestral MeoB system (8, 51, 52, 53).

First insights into the structural mechanism were revealed by the crystal structure of *Saccharomyces cerevisiae* UBA1 in complex with Ub (Schindelin, Cell 2008) (11). In the “adenylation-competent” conformation of UBA, the UFD adopts the distal conformation and the SCCH domain adopts the open conformation (Fig. 2, Step 1). This UBA1/Ub structure showed that Ub binding to UBA1 buries approximately 3200 Å^2^ of solvent exposed surface area through three distinct interfaces (11, 54). The first interface involves the highly conserved hydrophobic isoleucine (Ile44) patch of Ub interacting with β23 and β24 of the AAD, supported by additional hydrophobic interactions between β3 and β4 of Ub and α6 and α7 of the 4HB in the IAD. A polar contact between the carbonyl of Ub Leu8 and UBA1 Asn900 further stabilizes this interaction. The second interface stabilizes and guides the C-terminal tail of Ub, with Arg42, Arg72, and Arg74 forming key contacts with the crossover loop of UBA1. Among these, Arg72 plays a pivotal role in ensuring Ub specificity for UBA1 by interacting with a negatively charged pocket formed by the crossover loop (55). The third interface, between Ub and the FCCH domain, is primarily polar and varies across Ubls, being more extensive in NEDD8 but absent in SUMO (47, 56).

Together, these interfaces allow for the proper binding of Ub and the positioning of its C-terminal glycine (Gly-76), allowing the carboxylate of the C terminus to nucleophilically attack the α-phosphate of ATP. This reaction forms a mixed anhydride transition state, stabilized by the Mg^2+^ ion and the conserved aspartate residue (53). While several structures have captured UBA1 with Ub and ATP-Mg^2+^ bound prior to hydrolysis, the first structure of the Ub-AMP acyladenylate intermediate was captured by Schafer *et al.* in 2014 (PDB: 4NNJ) (57).

The adenylation mechanism of E1 enzymes has deep evolutionary roots, as exemplified by the bacterial sulfur-transfer proteins MoeB and molybdopterin-converting factor subunit 1 (MoaD). MoeB, a homodimer structurally homologous to the AD of eukaryotic E1s, activates MoaD, a small β-grasp fold protein structurally related to Ubls. These bacterial components will be discussed further in the context of newly identified E1-like enzymes. The structure of human UBA1 closely resembles the MoeB–MoaD–AMP complex solved by Lake *et al.* in 2001 (PDB: 1JWB), with Gly41, Arg73, Lys86, and Asp130 of MoeB corresponding to Ala478, Arg515, Lys528, and Asp576 of hUBA1 (53). Notably, a bound sulfate in the active site could indicate the position of the pyrophosphate (PPi) leaving group, as it occupies the site of γ-phosphate of ATP and is being stabilized by Arg57 and Arg515—an interaction also observed in MoeB-MoAD-AMP structures. These findings highlight the evolutionary conservation of the fold and catalytic machinery within the active ADs of E1 enzymes.

#### Ub thioester bond formation

As noted above, the SCCH domain of UBA1 adopts distinct conformational states during its catalytic cycle, transitioning through the multistep processes of adenylate formation, thioester bond formation, and transthiolation. These substantial conformational changes are linked to the hydrolysis of ATP-Mg^2+^ on adenylation of Ub. Following the release of PPi, the SCCH domain undergoes a 124° rotation, transitioning from an “open” to a “closed” conformation (Fig. 2, Step 2) (47). This critical conformational shift allows the active site to accommodate the repositioned SCCH domain and its active site cysteine (Cys603 in hUBA1), bringing the residues essential for thioester bond formation into proximity with the C terminus of Ub. The movement also disrupts regions of the AD, causing the N-terminal helices to become disordered.

Additional disordering occurs in the “cys-cap,” which may protect the catalytic cysteine from oxidative damage in the open conformation. In the closed conformation, the cys-cap becomes disordered, potentially to prevent steric clashes during active site restructuring and to enhance reactivity by increasing solvent accessibility (58). Collectively, these changes effectively switch the active site from an “adenylation-competent” to a “thioester-competent” state.

Early biochemical studies have shown that the rate-limiting step of adenylation is the release of PPi following the formation of the E1-AMP-Ub intermediate (10). This release is proposed to trigger the conformational changes that facilitate the formation of the E1-Ub thioester bond while also preventing the back reaction, as the adenylation-competent conformation has been disassembled (9). An additional level of enzymatic regulation is contributed by the local concentrations of PPi and AMP, both of which inhibit thioester formation as their concentrations increase (9).

After the active site cysteine of UBA1 attacks the adenylated Ub [Ub(a)], AMP is released, and the protein returns to the “open” conformation. In this state, the carbonyl carbon of Ub is linked to the active site cysteine (C632 in hUBA1) *via* a thioester bond (Ub∼E1, [Ub(t)]). This transition deconstructs the thioester-competent state and reforms the adenylation-competent state, allowing for the binding of an additional Ub and ATP-Mg^2+^, as observed in PDB: 4NNJ (Fig. 2, Step 3) (57). In this conformation, the AD holds a noncovalently bound Ub-AMP, while the SCCH domain carries a thioester-linked Ub. This double-loaded state is a key intermediate in the E1 catalytic cycle and enhances the efficiency of Ub transfer to the E2 enzyme. Structural and kinetic studies suggest that this state promotes the conformation best suited for E2 binding and facilitates the coupling of transthiolation with adenylation and PP_i_-Mg^2+^ release, contributing to the “burst” kinetics observed during E2 loading (59, 60).

In the double-loaded state, Ub(t) is displaced ∼31 Å from the Ub(a) position, with its most prominent interactions occurring between the β-grasp domain of Ub(t) and the FCCH domain. This forms a smaller interface with UBA1 (∼900 Å^2^) than the ∼1600 Å^2^ interface formed by Ub(a) with UBA1. The proximity of Ub(t) to the FCCH leaves sufficient space for the binding of an incoming E2 (48). The smaller buried surface area also suggests weaker interactions between Ub(t) and UBA1, which may facilitate the transfer of Ub(t) to E2 and their subsequent disassociation. However, as Ub(t) is covalently linked to the SCCH *via* a thioester bond, rapid disassociation prior to transthioesterification is prevented. In the process of transthioesterification, additional contacts are formed to Ub(t) from the E1 and E2, particularly those critical for stabilizing the tetrahedral transition state during transthioesterification (48, 61).

#### E1-E2 specificity, flexibility, and transthioesterification

There are approximately 40 E2s in humans, with the majority thought to be involved in Ub transfer (62, 63). Studies have shown that E2 specificity for an E1 is mediated by the C-terminal UFD of the E1, with chimeric E1 proteins (*e.g.*, UBA7 with the UFD of UBA1) priming different sets of E2s with their respective Ubl (46, 48, 57, 64, 65, 66, 67). Despite this specificity, E2s exhibit significant differences in their binding interfaces with the UFD, even while binding with similar affinities (56). This diversity necessitates a high degree of promiscuity in UBA1 without compromising its enzymatic activity. Although the specific residues involved in E1 recognition vary among E2s, the overall mechanism of E2 binding to E1 and the subsequent transthioesterification reaction is likely conserved.

Structural studies have provided critical insights into the interactions between UBA1 and its E2 partners, capturing several states during the catalytic cycle. As of this review, structures of UBA1 in complex with several E2s—Ubc4 (PDB: 4II2, 9B5N, 9B5C), Ubc13 (PDB: 6ZHS), Ubc15 (PDB: 5KNL) and Ubc3 (PDB: 6NYA) (46, 60, 65, 66)—have been solved. These structures sample the conformational states adopted by UBA1, Ub, and E2 during the catalytic process, providing valuable insights into the molecular mechanism of transthioesterification.

E2s initially bind to the UFD domain in a “distal” conformation relative to the SCCH domain (illustrated in Fig. 2, Step 3). This interaction is primarily mediated by salt bridges formed between residues on helix A (hA) of the E2 and a highly conserved acidic patch of the E1 UFD (64, 65). The hA of E2s is a key structural element of E2s that mediates interactions with the stable platform contributed by the UFD domain. A highly conserved three-residue basic motif [(K/R)RXX(K/R)] on hA facilitates the binding to the conserved acidic patch. Biochemical studies have demonstrated that loss of these residues results in a significant reduction in thioester transfer activity (46, 56, 68). The conformational plasticity of hA interactions with the acidic patch is critical for accommodating the structural diversity of cognate E2 enzymes, as the hAs of different E2s form distinct interfaces with the acidic patch of the UFD. As a result, superimposition of the E2s bound to the distal UFD shows that their catalytic cysteines occupy positions up to 7 Å apart, despite needing to perform the same transthioesterification reaction.

To accommodate the conformational plasticity in hA–UFD interactions, both the UFD and SCCH undergo conformational adjustments to bring the catalytic cysteines into proximity, with the UFD twisting up to ∼25° to adopt a proximal or “closed” position (Fig. 2, Step 4) (48). In this position, helices C and D establish contacts with residues surrounding the catalytic cysteine of the SCCH domain, and disruption of these contacts leads to moderate reductions in thioester transfer activity. A tripartite network of contacts also forms between the E2, Ub(a), and the UBA1 crossover loop, further stabilizing this conformation.

Additionally, residues contributed from the α2-α3 loop of the E2 and from Uba1 form the sides of a groove that guides the C-terminal residues 74-76 of Ub(t) to the E1/E2 active sites. Beyond the active site, a pocket is blocked by residues contributed from both the E1 and E2, effectively locking the Ub C terminus in place to enable catalysis. The α2-α3 loop is critical for thioester transfer but remains poorly ordered in structures until stabilizing intramolecular interactions form between an asparagine residue of the highly conserved HPN motif in E2s and residues within the α2-α3 loop. Additional van der Waals interactions occur between a conserved proline and a hydrophobic patch of the SCCH domain. Residues from the α2-α3 and UBA1 are in proximity to form the oxyanion hole that stabilizes the charged transition state, but it is not yet clear which residues would deprotonate the nucleophilic cysteine of the E2.

Following the thioester transfer, the contacts between Ub and E1 are disrupted as Ub transitions from an “open” to a “closed” conformation (Fig. 2, Step 5). This loss of interactions with the E1 is accompanied by a strengthening of contacts with the E2, facilitating the disengagement of Ub∼E2 complex from the E1. This transition has been visualized in the crystal structure of UBA1 with Ubc3 (Olsen *et al.*, 2021; PDB: 7K5J) as well as in cryo-EM structures of UBA1 with Ubc4 single- and double-loaded states (Lima *et al.*, 2024. PDBs: 9B5N *etc.*) (48, 60).

In the “starting” position, with the E2-bound UFD twisted toward the SCCH, Ub(t) occupies the same position observed in the doubly loaded UBA1 structure (PDB:4NNJ). Subsequent structures capture the transition of Ub(t) as it shifts from interacting predominantly with the FCCH to contacting the E2. During this transition, Ub(t) undergoes a 180° rotation and shifts ∼35 Å from its position near the FCCH to a location proximal to the SCCH.

Interestingly, the cryo-EM structure where Ub(t) is most proximal to the E2 (state 10, PDB: 9B5O) differs in Ub placement by a ∼55° rotation compared to the previously solved crystal structure (PDB: 7K5J). In 7K5J, the position of Ub(t) aligns with the structure of Ubc3-Ub(t) in the absence of UBA1 (PDB: 4MDK) (69). While it is important to note that the E2 is different between the structures (Ubc3 in the crystal structure *versus* Ubc4 in cryo-EM), the cryo-EM state may represent a conformation on the path to Ub(t)∼E2 disassociation. In contrast, the conformation captured in 7K5J may correspond to the state Ub(t) adopts immediately prior to or following disassociation.

Additional insights from the cryo-EM structures reveal that as Ub(t) undergoes its 180° rotation, density for PPi is lost from the adenylation active site. These observations suggest that Ub(A) adenylation and PPi release are coupled to transthiolation. Supporting this, biochemical data show that adding a nonhydrolysable Ub-AMP mimetic (Ub-AMSN) and PPi to UBA1∼Ub(t) promotes transthiolation more effectively than other reactants (*e.g.*, Ub(a) alone or with ATP-Mg^2+^). This indicates that the mimetic may stabilize the “adenylation-ready” conformation of UBA1, which in turn stabilizes the transthiolation-competent form of the SCCH. These findings align well with previous reports showing that doubly loaded UBA1 is more efficient at transthiolation (9). Once the UFD reopens, the Ub-E2 is released to interact with an E3 ligase (Fig. 2, Step 6). The Ub is subsequently transferred from the E3 ligase to a substrate, marking it for various downstream processes. This leaves the E1 enzyme free to form a new thioester bond with the second Ub(a), allowing the cycle to repeat.

**Figure 3:**
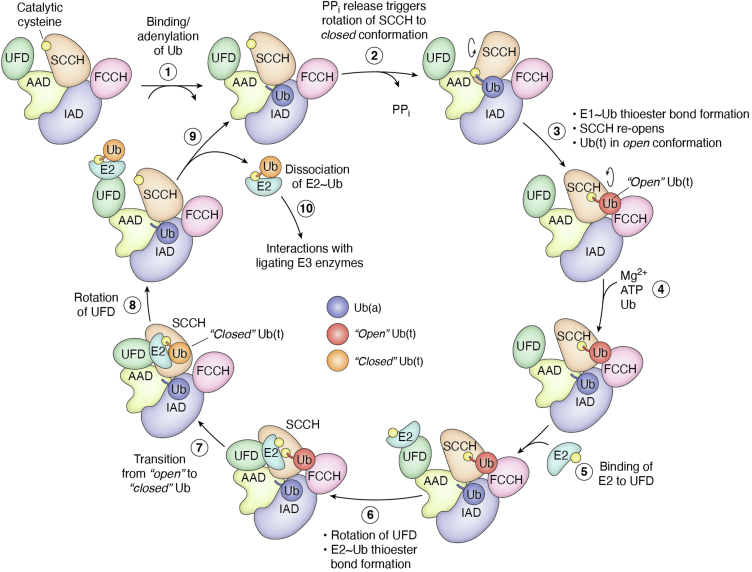
**Current model for the canonical E1 catalytic cycle.** Simplified schematic of the structural changes involved in the canonical E1 catalytic cycle. *1*, ATP-Mg^2+^ and Ubl bind, and the Ubl is adenylated. *2*, loss of PP_i_ triggers rotation of the SCCH to the closed conformation. *3*, E1∼Ub thioester bond formation occurs, SCCH reopens. *4*, a second set of ATP-Mg^2+^-Ub binds to the AAD. *5*, the E2 binds to the UFD. *6*, the UFD rotates toward the SCCH, bringing the catalytic cysteine of the E2 into proximity of the E1∼Ub thioester bond. *7*, contacts between Ub(t) and the FCCH domain are lost as new ones are formed between Ub(t) and the E2. *8*, the UFD returns to the “open” conformation. *9*, the Ubl(t)∼E2 disassociate, allowing for formation of a new thioester bond to the Ubl(a) bound to the AAD. *10*, the E2∼Ubl(t) are free to interact with ligating E3 enzymes. AAD, active adenylation domain; FCCH, first catalytic cysteine half-domain; PPi, pyrophosphate; SCCH, second catalytic cysteine half-domain; Ub, ubiquitin; Ubl, ubiquitin-like protein; UFD, Ub-fold domain.

All canonical E1s share a common catalytic cycle and a mechanism involving domain restructuring (summarized in Fig. 3). However, these enzymes have evolved specific protein–protein interactions that enable them to differentiate between their respective Ubls and E2s, as well as variations in domain structure and ligand-binding sites that modulate enzyme activity. These differences will be the focus of the following sections. Unless otherwise specified, the overall pattern of Ubl binding, adenylation, and thioester transfer will follow the mechanism previously described for UBA1.

### Ub/FAT10 E1 (UBA6)

UBA6 is an unusual E1 enzyme, sharing only 40% sequence homology with UBA1 but uniquely capable of activating both Ub and the Ubl FAT10 (58). Found exclusively in chordates and echinoderms, UBA6 likely emerged from a common ancestor of these phyla approximately 500 million years ago (70, 71). Rather than functioning as a backup for UBA1, UBA6 engages a distinct set of E2 enzymes and substrates, supporting nonredundant roles in regulating proteostasis, cytoskeletal dynamics, and immune signaling (70, 72, 73).

UBA6 is expressed broadly but is particularly important in the brain and immune system. Its disruption leads to severe physiological consequences: complete knockout in mice causes embryonic lethality or developmental arrest, while neuronal-specific deletion results in synaptic defects, behavioral abnormalities and impaired neurodevelopment (74, 75, 76, 77, 78). In humans, biallelic mutations in UBA6 have been linked to neurodevelopmental disorders, including intellectual disability and autism spectrum disorder—likely due to defective Ub signaling and synaptic protein turnover (79, 80). Altered expression of UBA6 has also been observed in several cancers, and its selective activity is under investigation as a potential therapeutic target (58, 81, 82, 83, 84). As the only other E1 enzyme capable of activating Ub, UBA6-specific activation of Ub plays an indispensable and specialized role in maintaining cellular homeostasis, with critical implications for brain function.

UBA6 and its cognate E2, UBE2Z, are the only known E1-E2 pair capable of catalyzing FAT10 conjugation (85). FAT10, a two-domain protein composed of tandem Ub-like folds, marks substrates for rapid proteasomal degradation. Unlike Ub, which is cleaved and recycled prior to substrate degradation, FAT10 is degraded along with its substrate (86). Although UBA6 is essential for embryonic development, FAT10 itself is not, as FAT10 KO mice are viable with mild metabolic defects (87, 88). FAT10 expression is strongly induced by inflammatory stimuli *via* NF-κB signaling (89, 90, 91) and upregulated during viral infection through TNF-α and INF-γ (92, 93, 94, 95). Overexpression of FAT10 is linked to chromosomal instability and can trigger apoptosis (96, 97, 98), while its dysregulation contributes to tumorigenesis (99, 100, 101, 102).

#### A conserved binding pocket for allosteric regulation

**Figure 4:**
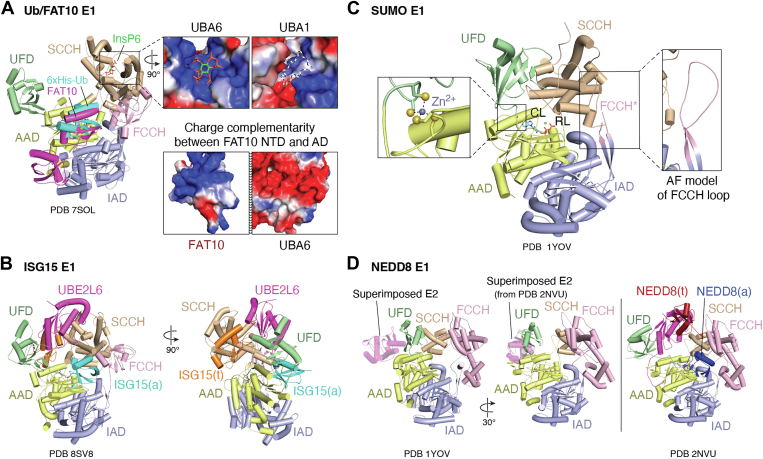
**Structural features of the FAT10, ISG15, SUMO, and NEDD8 E1s.***A*, comparisons of ubiquitin (Ub, PDB: 7SOL) and FAT10 (from PDB: 7PYV, superimposed) binding at the adenylation site of UBA6. The *top inset* highlights the InsP_6_ binding pocket in UBA6 and the corresponding region in UBA1, where a transparent InsP_6_ molecule is modeled for comparison. The *lower panel* shows the surface electrostatics between the NTD of FAT10 and the AD of UBA6, with surface charges displayed using a consistent scale across all images. *B*, cryo-EM structure of the UBA7–UBE2L6–ISG15 complex, illustrating the unique position of ISG15(t) at the back of the enzyme. The NTD domains of ISG15 are not modeled in the structure. *C*, a catalytically important zinc binding motif (*left*) is formed between two cysteines from the crossover loop and two from the loop leading to the UFD. The FCCH domain is significantly reduced in the SUMO E1. An AlphaFold model of the loop is shown to the *right*. *D*, *left*: superimposition of E2-UFD from the APPBP1-UBA3∼NEDD8-NEDD8-MgATP-Ubc12 crystal structure (PDB: 2NVU) onto the PDB of apo NEDD8 E1 (PDB: 1YOV). *Middle*: the same superimposed model rotated ∼30° to visualize domain orientations. *Right:* structure of the complete NEDD8 E1–E2–Ubl complex (PDB: 2NVU) showing the bound E2 and the NEDD8 Ubl in the thioester transfer-competent conformation. AD, adenylation domain; InsP_6_, inositol hexakisphosphate; ISG15, interferon-stimulating gene 15; NEDD8, neural precursor cell expressed developmentally downregulated protein; NTD, N-terminal domain; SUMO, small ubiquitin-like modifier; UBA, ubiquitin-activating enzyme; Ubl, ubiquitin-like protein; UFD, Ub-fold domain.

As a canonical E1, UBA6 shares the same domain structure and catalytic cycle as UBA1 (Fig. 4*A*) (58). Crystal structures have captured the SCCH domain of Ub-bound UBA6 transitioning from an adenylation-competent to a thiolation-competent conformations, involving a ∼136° rotation of the SCCH (PDB: 7SOL) (58). Unexpectedly, these structures also revealed that inositol hexakisphosphate (InsP_6_) binds to a highly conserved basic pocket in the SCCH, located proximal to the catalytic cysteine (Fig. 4*A*, top left inset). Notably, InsP_6_ was not included in the crystallographic conditions but had copurified with UBA6 during insect cell protein expression. Subsequent isothermal titration calorimetry experiments confirmed that InsP_6_ binds to UBA6 with nanomolar affinity, while no binding of InsP6 to UBA1 was detected. This is consistent with the absence of an equivalent, well-conserved binding pocket for InsP_6_ in UBA1 (Fig. 4*A*, top right inset).

Interestingly, InsP_6_ forms several contacts within the SCCH that become disordered or are involved in conformational changes during the transition to the thioester-competent active site. These observations suggest that InsP_6_ modulates UBA6 activity by stabilizing the “open” conformation, inhibiting the rotation of the SCCH and thereby reducing catalytic efficiency. Given that InsP_6_ concentrations in eukaryotic cells are within the 10 to 100 uM range—well above the measured Kd—this interaction is likely physiologically relevant. It may also explain in part why UBA6 exhibits only 50% of the activity of UBA1 in proliferating HEK 293T cells and accounts for just ∼1% of total Ub activation (70, 103).

#### Dual specificity for Ub and FAT10

To activate both Ub and FAT10, UBA6 must accommodate both proteins at its active site, despite the C-terminal domain (CTD) of FAT10 sharing only 36% sequence identity with Ub and just 31% similarity in residues that contact the UBA6-binding site (58, 104). Ub binding to UBA6 mirrors its interaction with UBA1, with hydrophobic interactions between the Ile44 patch of Ub and the AAD domain of UBA6. In contrast, FAT10 lacks an equivalent hydrophobic patch and instead features a divergent “polar patch,” requiring a distinct set of interactions to stabilize its binding (Fig. 4*A*, bottom insets).

A chimeric version of UBA6, in which the SCCH domain is replaced with that of UBA1 to improve stability, was used to solve a crystal structure of UBA6 with FAT10 bound in the adenylation site (72). As FAT10 consists of two tandem Ub-like folds, both domains are utilized to bind UBA6. Surprisingly, the binding surface for FAT10 (∼1700 Å^2^) is only slightly larger than that of Ub (∼1600 Å^2^), despite FAT10’s dual Ub-like domains. The CTD of FAT10 forms a relatively smaller interface with UBA6 than Ub, with additional contacts contributed by interactions of the N-terminal Ub-like fold with the 4HB of the IAD. Both Ub and FAT10 C-terminal bind to the same hydrophobic section of the AAD. However, the loss of these hydrophobic contacts effect FAT10 binding less significantly than it does Ub, likely due to the stabilizing contributions from FAT10’s N-terminal domain (NTD).

The last six residues of FAT10 (Cys-Tyr-Cys-Ile-Gly-Gly) differ significantly from those of Ub (Leu-Arg-Leu-Arg-Gly-Gly). As C-terminal residues—particularly R72 of Ub—are known to be key determinants of Ubl binding specificity, it was unclear how UBA6 maintains specificity for both proteins. In the chimeric UBA6 structure, the pocket that recognizes R72 of Ub was shown to also accommodate Y161 of FAT10. However, the interactions with Y161 are predominantly hydrophobic, in contrast to the polar side chains that stabilize R72. Notably, mutations of polar residues in this site were found to impair activation of Ub without affecting FAT10 activation.

These findings suggest potential strategies for the selective inhibition of UBA6’s activity toward a single target, enabling deconvolution of its Ub and FAT10 functions both *in vitro* and *in vivo*, as well as assessing the effects of lowered activation on cell viability. Further studies are needed to validate FAT10 interactions using full-length UBA6 (with and without bound InsP_6_) and to elucidate the binding surfaces of Ub and FAT10 during transthiolation to an E2. Of particular interest would be identifying the interfaces formed between UBA6 and FAT10 prior to transthiolation, and how UBE2Z discriminates between Ub and FAT10 to maintain specificity for FAT10.

Notably, Parkin, a well-characterized E3 ligase of Ub, was recently identified as the first E3 enzyme to facilitate FAT10ylation (105). This is significant as autosomal recessive mutations in Parkin are a known cause of early onset Parkinson’s disease. Parkin has been shown to autoFAT10ylate itself *in vitro*, targeting it for proteasomal degradation. This action may inhibit mitophagy of damaged mitochondria, leading to the generation of reactive oxygen species that may result in neuronal apoptosis, a hallmark of early-onset Parkinson’s disease (106).

### ISG15 E1 (UBA7)

UBA7 is the sole E1 for interferon-stimulating gene 15 (ISG15), a key component of the innate immune response expressed downstream of viral induced signaling pathways and the second Ubl to be discovered after Ub (61, 64, 107). ISG15 conjugation is canonically known to inactivate viral proteins, modulate components of the innate immune system, and promote inflammation through extracellular ISG15 acting in a cytokine-like manner (108). Furthermore, ISG15 has been shown to regulate cellular signaling, modulate proteasomal activity, influence autophagy, and inhibit mRNA translation (109). UBA7 transfers ISG15 to its specific E2, UBE2L6, which facilitates ISG15 conjugation to target proteins *via* the E3 ligases HERC5, HHARI, TRIM25, and potentially others (110, 111).

Surprisingly, mice lacking UBA7—and therefore incapable of ISGylation—are viable, fertile, and display no overt phenotypic abnormalities (112, 113). Although ISG15 conjugation is strongly induced by viral infection, UBA7-deficient mice show no significant different from controls upon infection with vesicular stomatitis and lymphocytic choriomeningitis viral infections, suggesting redundancy within interferon signaling pathways. However, ISGylation appears to play a role in immune surveillance, as ISG15 suppresses breast tissue growth and metastasis, implicating it in cancer detection and immune response (114). Altered ISGylation levels have also been linked to human disease (109). Notably, ISG15 deficiency is associated with severe mycobacterial infections (115, 116), and increased ISGylation has been observed in peripheral blood mononuclear cells from Coronavirus Disease 2019 patients (117), highlighting a critical role for ISG15 in host defense.

#### Conserved architectures with variable surfaces

UBA7, like UBA1 and UBA6, is composed of a pseudosymmetric base with FCCH and SCCH insertions and a C-terminal UFD (Fig. 4*B*). Structures of UBA7 in complex with UBE2L6 and ISG15 have been solved, with results mostly consistent with what has been previously observed with UBA1 and UBA6. ISG15, like FAT10, consists of an N-terminal and C-terminal Ub-like domain. The CTD of ISG15 binds at the AAD, forming a continuous interface of ∼1500 Å^2^ (64). As with Ub, the interface is composed of three distinct interfaces.

The first is a polar interaction network with the AAD, termed the “polar patch,” which contrasts with the hydrophobic Ile44 patch seen in Ub. While single mutations in this region have modest effects on activity, multiple charge-disrupting mutations significantly diminish ISG15 activation (61). Surrounding this polar patch, additional hydrophobic contacts further stabilize binding. The second interface consists of salt bridges and hydrogen bonds between the side of the β-grasp domain and the FCCH domain of UBA7. The third interface involves residues from the AAD and crossover loop of UBA7 and resembles the interactions seen with UBA1-Ub, as ISG15 and Ub share identical C-terminal sequences (LRLRGG). This is notable as residue R72 of Ub serves as a well-established specificity filter for E1–Ubl interactions (55). For ISG15, however, specificity appears to arise from the sum of its interfaces with the AAD. Mutational studies by Swatek *et al.* showed that replacing all residues in the ISG15 C-terminal tail involved in the ISG15/AAD interface with Ub equivalents enabled misactivation of ISG15 by UBA1 and transfer to UBA1-specific E2s, without significantly affecting activation by UBA7 (118). These findings underscore the complexity of Ubl-E1 specificity, demonstrating that multiple mechanisms contribute to selectivity.

In all available structures of UBA7, the NTD of ISG15 was poorly represented in cryo-EM maps and thus was not included in the final structural models. Weak resolution data hints that the NTD of ISG15(a) can extend toward and contact the UFD of UBA7, potentially acting to promote the “proximal” conformation of the UFD that aids thioester transfer. However, the lack of clear density indicates an inherent flexibility for the NTD, suggesting that the contacts it makes with UBA7 are not critical for activation and thioester transfer. This is further supported through mutational work that demonstrates comparable rates of ISG15 transfer despite deletion of the entire NTD (118).

Additional work with the influenza B virus NS1 protein (NS1B) show that NS1B prevents conjugation of ISG15 to substrates by binding to the N terminus, but not to the C terminus. Time course thioester transfer assays with full-length ISG15 coincubated with NS1B were also shown to occur at approximately the same rate. Together, these studies indicate that the C terminus is critical for specificity and activation, while the N terminus has a role in substrate conjugation (119).

#### Thioester transfer from a unique position

As mentioned earlier, recruitment of the ISG15-specific E2, UBE2L6, is mediated through interactions with the UFD (64). This was demonstrated in the 2008 study by Durfee *et al.*, where the creation of chimeric E1 and E2 variants (*e.g.*, UBA7 with a UBA1 UFD) facilitated the preferential transfer of ISG15 to the closely related E2, UBE2L3 (64). Residues 1-39 of UBE2L6, corresponding to hA and β1-β2 of the E2, were identified as critical for specificity. The study raised an intriguing question: why has not further divergence in E2 sequence and structure occurred? One hypothesis suggested constraints imposed by HECT E3 recognition, limiting how divergent an E2 can become. Alternatively, the system’s fine-tuning may mean that even minimal mutations can have outsized effects on affinity, precluding an evolutionary pressure for further divergence.

A surprising feature of the doubly loaded UBA7 structure is the positioning of ISG15(t). While ISG15(a) is located on the “front” of the enzyme, consistent with other canonical E1s, ISG15(t) is located on the back of the enzyme, with its CTD sandwiched between the UFD and SCCH (Fig. 4*B*) (61). Notably, distinct “open” and “closed” conformations of ISG15(t) are not observed, making the UBA7-ISG15-UBE2L6 system an outlier compared to other canonical E1s. Structural analysis and modelling of ISG15 in hypothetical open or closed conformations suggest that either position would result in steric clashes between the NTD of ISG15(t) and either ISG15(a) or UBE2L6. Additionally, the divergent “polar patch” of ISG15 lacks the contacts with the crossover helix of UBE2L6 that are consistently observed between the hydrophobic patch of Ub and its cognate E2s in available structures. Together, these findings highlight the positioning of ISG15(t) on the back of UBA7 as a distinctive feature of this E1 system.

### SUMO E1 (UBA2/SAE1)

The reversible modification of predominantly nuclear proteins by small ubiquitin-like modifier (SUMO) is an essential cellular process that regulates protein stability, cell division, and DNA repair (120). Humans express three functional SUMO isoforms (SUMO-1, SUMO-2, and SUMO-3), each producing distinct downstream effects (121). SUMO-1 shares 43% sequence identity with SUMO-2 and SUMO-3, which are nearly identical (96% sequence similarity) and are often referred to collectively as SUMO-2/SUMO-3. The most abundant isoform in mammals is SUMO-2, and it has been shown to be required for mouse embryonic development, whereas SUMO-1 and SUMO-3 were not essential (122). There are many more copies of SUMO-1 and SUMO-2 coding regions in the human genome, but these pseudogenes are not capable of expressing functional isoforms of the Ubl (120).

Knockout of the SUMO E1 components (SUMO E1 enzymes [SAE1 or SAE2]) is embryonic lethal across multiple model organisms. In zebrafish, a null mutation in *uba2* (SAE2) causes morphological defects and complete lethality by 12 days postfertilization, while *uba2* loss in *C. elegans* similarly results in embryonic death (123, 124). SUMO E1 activity is also essential for hematopoietic progenitor cell maintenance, and its loss leads to a collapse in blood development (125). Although somatic mutations in humans are rare, biallelic missense mutations in *sae2* cause aplasia cutis congenita with ectrodactyly skeletal syndrome, a disorder characterized by growth retardation, dysmorphic facial features, neurodevelopmental delay, skeletal abnormalities (ectrodactyly, dysplasia, scoliosis), and internal organ defects (123, 126). Dysregulation of SUMO E1 has also been implicated in cancer. Overexpression of SAE1/SAE2 correlates with poor prognosis in breast and hepatocellular carcinomas (127, 128), while genetic knockdown halts proliferation of lymphoma cells *in vitro* and suppresses tumor growth in mouse xenograft models (129, 130, 131). These findings highlight the therapeutic potential of targeting SUMO E1, a topic explored in more detail in a later section.

Structurally, the SUMO fold closely resembles Ub, despite sharing only 18% sequence identity (132). A key difference is an unstructured N-terminal extension found in all SUMO orthologs. Surprisingly, rather than assist the interaction of SUMO and proteins with SUMO interacting motifs, these intrinsically disordered regions were found to inhibit said interactions by forming transient complexes with the SUMO-interacting motif–binding groove of SUMO (133, 134). This inhibitory effect provides an additional layer of control for the downstream effects of SUMOylation.

#### Heterodimer with zinc-binding motifs

The SAE is a 113 kDa pseudosymmetric heterodimer of SAE1 and SAE2, making it the first canonical E1 heterodimer discussed here. Although its overall domain structure resembles that of other canonical E1s, the FCCH domain is markedly reduced to a single disordered loop (Fig. 4*C*) (135). SAE1 contains the IAD and the FCCH loop, whereas SAE2 contains the AAD, SCCH, and the UFD. SUMO binding buries approximately 20% of its surface (∼1650 Å^2^), and contacts are exclusively formed with the SAE2 subunit. There does not appear to be any kinetic differences between the human SUMO isoforms *in vitro*, despite some differences in the sidechains that form contacts with SAE2.

A surprising structural feature of SAE is a conserved zinc-binding motif formed by four cysteines, two in the SAE2 crossover loop and two in the re-entry loop before the polypeptide terminates in UFD domain (Fig. 4*C*, left inset) (135). This zinc-binding domains links the CCH and UFD domains, but the bound Zn^2+^ was not observed to make contacts with either SUMO or ATP and deletion of one of the residues did not significantly impair E1∼thioester conjugation. Subsequent studies demonstrated that the zinc motif (as well as residues adjacent to the motifs) were critical for SUMO binding, as mutation of these residues prevented the E1∼SUMO thioester bond (136). Because these interactions were not visible in the initial crystal structures, it is likely that the zinc-coordinating motif contributes lower affinity binding surfaces that nonetheless remain essential for SUMO association.

As with other pseudosymmetric, contiguous E1 enzymes, the SAE1/SAE2 complex undergoes significant conformational rearrangement during catalysis (67, 137). After SUMO is bound and adenylated, the catalytic cysteine domain of SAE2 rotates approximately 130°, restructuring the active site into a thiolation-competent form. These changes deconstruct the adenylation-competent active site and the cysteine-containing helix (H6, residues 172-176 in human SAE1) to bring the cysteine toward the C terminus of SUMO for nucleophilic attack, with the transition state stabilized by an oxyanion hole formed by H6 of SAE2. Following thioester bond formation, the CCH returns to an open conformation, reforming the adenylation-competent active site to once again bind ATP-Mg^2+^ and SUMO.

#### Rotating the UFD

SUMO has a single dedicated E2, Ubc9 (138, 139, 140, 141). However, a structure of SAE in a transthiolation-competent conformation (*e.g.*, where the catalytic cysteines of Ubc9 and SAE2 are in proximity for thioester transfer) is not currently available. Modeling of Ubc9 onto the proposed E2-binding site of the UFD results in structures where the catalytic cysteine of Ubc9 is facing the opposite direction from the catalytic center of SAE (142). As complex domain rearrangements have been seen with each of the canonical E1s, it is likely that there is a similar mechanism underlying the positioning of the SUMO E2 that will be revealed as new structures are solved. Intrinsic affinity has been detected between Ubc9 and the catalytic cysteine domain of SAE, and these interactions may serve to guide the E2 into the proper configuration as the enzyme transitions to a transthiolation-competent state (143). Following transfer of SUMO to Ubc9, SUMO can either be conjugated to substrates directly by the Ubc9 or in tandem with one of the nine human genes determined to encode a SUMO-specific E3 ligase (144, 145).

### NEDD8 E1 (UBA3/APPBP1)

Neural precursor cell expressed developmentally downregulated protein 8 (NEDD8) is the Ubl with the highest sequence similarity to Ub (58%) and is best known for its role in regulating cullin RING ligases (CLRs) (146). Neddylation of CLRs, the largest family of Ub E3 ligase, trigger conformational changes that disrupt binding of cullin-associated and NEDDylation-disassociated 1 protein (147). The loss of cullin-associated and NEDDylation-disassociated 1 protein inhibition activates CLRs, and thus neddylation plays a critical role in Ub-dependent proteasomal degradation (148). Although noncullin substrates of NEDD8 have been reported, their physiological relevance remains controversial, in part because many findings rely on overexpression systems and the inherently transient nature of this post-translational modification (149, 150, 151).

NEDDylation is essential for early development, as knockout of either NEDD8 or its E1 enzyme is embryonically lethal in mice (150). Tissue-specific deletions further underscore its importance: liver-specific loss of NEDD8 or NEDD8 E1 leads to spontaneous fatty liver, cellular senescence, and neonatal death (152); NEDD8 E1 deletion in neural progenitor cells disrupts brain development and causes perinatal lethality (150); and heart-specific NEDDylation loss results in cardiac developmental failure (153, 154). In humans, NEDD8 E1 deficiency has been linked to congenital syndromes marked by severe intellectual disability, skeletal abnormalities, lymphopenia, and neurodegeneration (155). Conversely, NEDD8 overexpression and hyper-NEDDylation are associated with poor prognosis in several cancers, including breast, colorectal, and bile duct carcinomas (150, 156, 157).

#### Extended FCCH, reduced SCCH

NEDD8 is activated by the NAE1/NAE2 heterodimer. As in SAE1/SAE2, NAE1 contains the IAD and FCCH domains, while NAE2 harbors the AAD, SCCH and UFD domains. In contrast to SAE (and other canonical E1s), the FCCH domain is significantly expanded, whereas the SCCH is reduced (Fig. 4D). Due in part to the extended FCCH, NEDD8 binding buries approximately 3350 Å^2^ of solvent-exposed surface, 150 Å^2^ more than seen with the structurally equivalent Ub (11, 54, 158). Three main contacts between NAE and NEDD8 are formed: these are polar interactions between the acidic face of NEDD8 and the extended FCCH domain of NAE1, interactions between the conserved hydrophobic patch of NEDD8 and the AAD of NAE2, and contacts between the C-terminal tail of NEDD8 and NAE2’s AAD and crossover loop.

A critical determinant of specificity between Ub and NEDD8 is residue 72, an Arg in Ub, Glu/Gln in SUMO, and Ala in NEDD8. In NEDD8, Ala72 interacts with hydrophobic residues (specifically Leu206 and Tyr207 of NAE2), and mutation of either residue to the charged equivalents seen in either UBA1 or SAE greatly reduces NEDD8 adenylation rates (55, 158). Moreover, a NEDD8 A72R mutation abolishes adenylation by NAE, whereas changing Ub Arg72 to Ala permits Ub activation by NAE.

At the time of this writing, no published structure shows the catalytic cysteine of the NAE2 SCCH domain adopting a “closed” conformation for thiolation of the NEDD8 adenylate. Instead, the catalytic cysteine remains approximately 30 Å away from the active site in available structures. However, based on observations in other canonical E1 enzymes, it is likely that NAE2 undergoes a similar restructuring to position its catalytic cysteine for nucleophilic attack. Indirect evidence of this is seen in the 2010 work by Brownell *et al.*, where the E1 inhibitor MLN4924 forms an adduct with NEDD8 linked *via* thioester bond to the catalytic cysteine, thereby forming a NEDD8-AMP mimetic (159). Additional studies are needed to determine how these conformational changes occur.

#### Specificity through cryptic binding patches

NEDD8 conjugation occurs *via* two known E2 enzymes: UBE2F and UBE2M (160, 161). In *apo* and singly loaded NAE structures, the UFD’s E2-binding site orients the catalytic cysteine of a bound E2 away from the NAE catalytic cleft, consistent with previous modeling of SAE and Ubc9. In doubly loaded NAE structures, however, the UFD disengages from the AAD and rotates, exposing a portion of the AAD near the ATP-binding site. This forms a cryptic E2-binding site composed of the hydrophobic surface of the UFD along with the newly exposed AAD surface (PDB: 2NVU) (162). Furthermore, the N-terminal extensions of UBE2M binds into a groove on the AAD surface, an interaction that contributes to UBE2M’s specificity for the NEDD8 E1, alongside several surface residues that are critical for the efficiency of thioester transfer (162, 163, 164).

## Noncanonical E1s

Unlike canonical E1s, the noncanonical E1s (UBA4, UBA5, and ATG7) lack distinct catalytic cysteine half-domains or a Ub-fold domain. Instead, they more closely resemble their bacterial predecessors, employing distinct mechanisms to activate and transfer their respective Ubls. Despite their structurally simplified architectures, these E1s regulate essential cellular processes, and structural and mechanistic studies have revealed unexpected strategies by which they accomplish their functions.

### URM1 E1 (UBA4)

The Ubl Urm1 and its activating enzyme UBA4 are required for the nearly universal 2-thiolation of wobble uridines (s^2^U_34_) in tRNAs (165). The strong evolutionary conservation of this modification suggests it plays a critical role in translation fidelity, efficiency, and protein folding dynamics (166, 167). Loss of U_34_ thiolation disrupts cellular homeostasis and has been implicated in responses to heat shock and DNA damage (168). Notably, elevated temperatures can lead to a reversible loss of s^2^U_34_ modification in yeast through impairment of the URM1 pathway (169). Although UBA4 deficiency is rare, it can lead to severe disease. Reported cases include a missense mutation causing intellectual disability with Marfan syndrome–like features, and another involving profound neurological deterioration and early infant death (170, 171).

**Figure 5:**
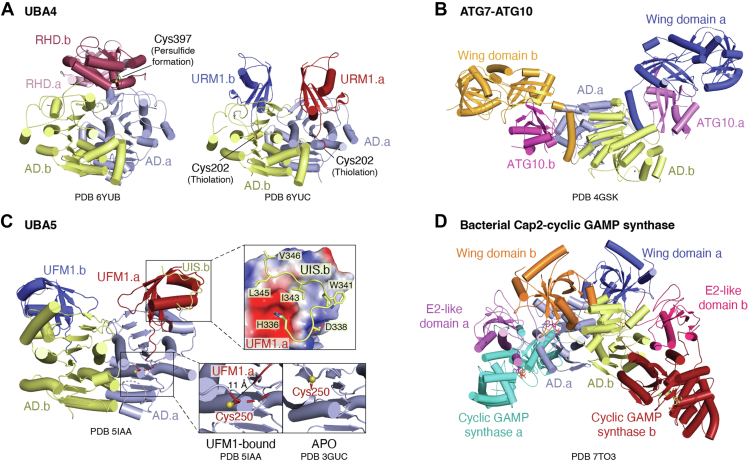
**URM1, UFM1, and ATG8/ATG12 E1s.***A*, comparison of *apo* (PDB: 6YUB) and URM1-bound (PDB: 6YUC) UBA4. The rhodanese homology domain (RHD) of UBA4 disengages from the adenylation domains in order to accommodate URM1. *B*, structure of the UFM1-bound UBA5 (PDB: 5IAA). *Top inset*: the UFM1-interacting sequence (UIS) forms hydrophobic and polar contacts to bind the UFM1 that is bound to the adenylation domain of the opposite UBA5 subunit. *Bottom insets*: the helix containing the active site cysteine (Cys250) melts on UFM1 binding to bring the catalytic cysteine closer to the C terminus of UFM1. *C*, structure of *S. cerevisiae* ATG7(E1)-ATG10(E2) crosslinked complex. *D*, structure of *Enterobacter cloacae* Cap2-DcnD02 2:2 complex. ATG, autophagy-related protein; Cap2, cGAS/DncV-like nucleoidyltransferase–associated protein 2; UBA, ubiquitin-activating enzyme.

UBA4 is a two-domain protein comprising an N-terminal AD and a C-terminal rhodanese-like domain (RHD), both of which are essential for the activation and thiocarboxylation of Urm1 (Fig. 5*A*) (172, 173). Structural studies have shown that UBA4 forms an asymmetric homodimer, with *apo* structures positioning both RHD domains on one side of the complex (165). Like canonical E1s, recent studies have revealed that Urm1 activation involves conformational rearrangements around a static AD to perform its catalytic activity.

**Figure 6:**
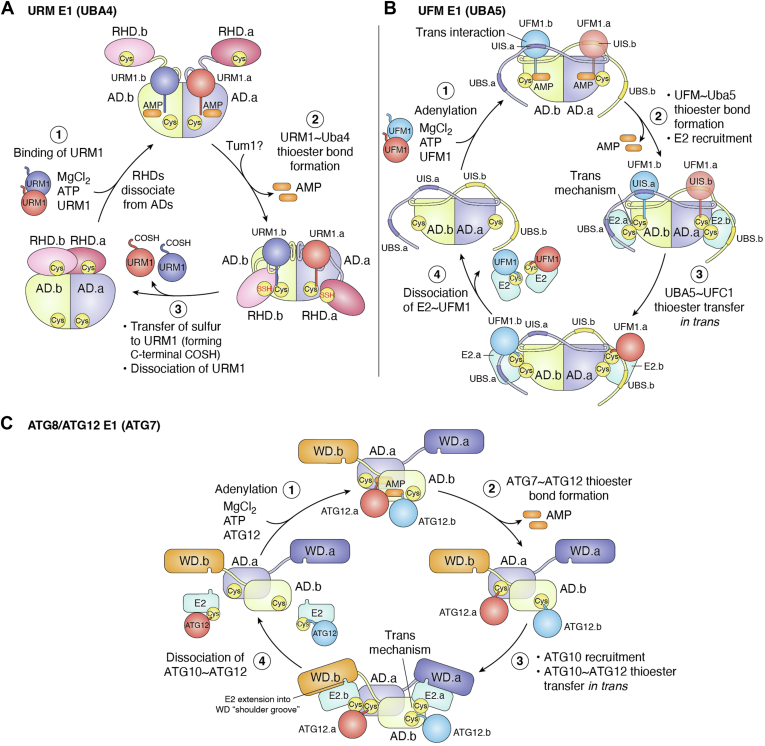
**Current models for the catalytic cycle of noncanonical E1s.***A*, URM1 E1 (UBA4): Upon binding of URM1 to the adenylation domains (AD1 and AD2), the rhodanese-homology domains (RHD1 and RHD2) disassociate from the adenylation core. URM1 is then adenylated, and a thioester bond forms between the catalytic cysteine of the AD active loop and the C terminus of URM1. It is hypothesized that persulfide formation on the RHD cysteine, mediated by Tum1, occurs while the RHDs are disassociated. The sulfur is subsequently transferred to URM1, generating Urm1-COSH, which is released. *B*, UFM1 E1 (UBA5): UFM1 binds to the adenylation domain and engages the UFM1-interacting sequence (UIS) of the opposite protomer in a *trans*-binding mechanism. A thioester bond is formed between the catalytic cysteine of UBA5 and UFM1 and the E2 enzyme UFC1 is recruited *via* the UFC1-binding sequence (UBS). UFC1 receives UFM1 through transthiolation, and the resulting UFM1∼UFC1 complex then disassociates. *C*, ATG8/ATG12 E1 (ATG7): ATG8 or ATG12 binds and is adenylated. The catalytic cysteines of ATG7 attack the Ub-adenylate to form a thioester linkage. The respective E2 (ATG3 or ATG10) is recruited by the wing domain of the opposite ATG7 protomer, and the E2 receives the Ubl *via* transthiolation. The Ubl∼E2 conjugate then disassociates from the ATG7 homodimer. ATG, autophagy-related protein; Ub, ubiquitin; UBA, ubiquitin-activating enzyme.

In its *apo* form (PDB: 6YUB), the RHDs of UBA4 appear inhibited, with the dimer interface of RHD_1_ and RHD_2_ sequestering the loops that contain the active cysteines. In the Urm1-bound structure (PDB: 6YUC), the RHDs become disordered and are not visible in the crystal density, suggesting that Urm1 binding releases the catalytic cysteines of the RHDs (Cys397 in *Tetrahymena thermophila*) from the dimer interface. Although it is not clear from available data at which step persulfide formation of Cys397 occurs, disruption of the RHD dimer may allow for thiolation by tRNA thiouridine modification protein (174). Meanwhile, in the adenylation active site, the active cysteine of the AD (Cys202) attacks adenylated Ufm1, forming a thioester bond. The RHD then repositions adjacent to the Cys202-Urm1 thioester, allowing the persulfide group on Cys397 to attack, forming the disulfide-linked E1-S-S-Urm1 intermediate. This reaction results in the release of Urm1-COSH, either autonomously or upon attack by thioredoxin peroxidase Ahp1 (175). Collectively, these data suggest a prokaryotic-like mechanism in which the adenylation and thioester linkage of the first Urm1 primes the enzyme for the binding of a second Urm1 at the other AD, potentially triggering rearrangements that facilitate Urm1-COSH release (Fig. 6*A*).

Following the formation of Urm1-COSH, the Ubl binds to the sulfur–transferase complex (CTU1/CTU2 in humans), selectively recruits tRNAs and then thiolates them (176, 177, 178, 179, 180, 181). Despite the evidence of Urm1 conjugation to proteins during oxidative stress, a dedicated E2 or E3 has yet to be discovered (181). Rather, Urm1-COSH has been shown capable of modifying proteins without a specific ligase, persulfidating available cysteine residues in an oxidative environment (182). It remains possible that an undiscovered group of “urmylases” exists that would result in transient Ubl-like modification/persulfidation of substrate proteins as an area of further study.

### UFM1 E1 (UBA5)

While UBA4 is considered the eukaryotic E1 most similar to prokaryotic precursors, UBA5 represents the most minimalist functional E1, capable of carrying out adenylation and thioester transfer despite being only ∼400 amino acids long, less than half the size of canonical E1s (∼1000+ residues) (183). UBA5 exists in two isoforms: a truncated splice variant (residues 57-404 of hUBA5) and a full-length form. Although the poorly conserved N-terminal extension is not required for UFM1 activation, studies indicate that it enhances ATP binding affinity and promotes UFM1 transfer to its cognate E2, UFC1 (184).

UBA5 activates UFM1, a Ubl that shares only 16% sequence identity with Ub but retains the conserved β-grasp fold common to Ubls (185). Uniquely, UFM1 terminates in a Val-Gly motif instead of the typical Gly-Gly found in most Ubls. UFM1 conjugation has been linked to several critical cellular processes, including the unfolded protein response, endoplasmic reticulum stress, autophagy, DNA repair, and immune response (186, 187, 188, 189, 190, 191). Interestingly, UBA5 knockout in mice does not result in early embryonic lethality but causes severe anemia and death *in utero*, highlighting a critical role in erythroid differentiation (192). Dysregulation of the UFM1 cascade is associated with multiple human diseases, including Alzheimer’s disease (193), hematopoietic disorders (194), chondrodysplasias (195), and cancers (196, 197).

#### The minimalists’ E1

Structural studies have shown that UBA5 functions as an obligate homodimer, utilizing a unique *trans*-binding mechanism to bind UFM1 with an ∼70 amino acid C-terminal extension (Fig. 5*B*) (198, 199, 200). Specifically, the β-grasp domain of UFM1 interacts with the AD of one subunit and the UFM1-interacting sequence of the opposite subunit. UFM1 binding induces conformational changes in UBA5, particularly at the crossover loop containing the catalytic cysteine (Cys250 of hUBA5). This loop, structured as an α-helix in *apo* UBA5, becomes a flexible loop in the UFM1-bound state (PDBs: 3GUC, 5IAA), mirroring the mechanisms observed in other E1s, where secondary structure melting is necessary to position the active site cysteine. Deletion of the C-terminal extension prevents UFM1 activation, and mutations disrupting the *trans*-binding contacts inhibit UFM1 activation (Fig. 6*B*).

The *trans*-binding mechanism of the C-terminal extension also mediates UFC1 recognition and binding, with residues 392-404 forming a UFC1-binding sequence (UBS) (198). This UBS interacts with a hydrophobic pocket on UFC1 that is notably absent in other E2s, reinforcing its specificity for UBA5 (PDB: 7NW1) (201). Additionally, this interaction appears to enhance UFM1 transfer; UFC1’s active cysteine is more solvent-exposed than in other E2s, and binding of the UBA5 UBS may promote desolvation of the active cysteine, increasing its nucleophilicity for transthiolation. Once UFM1 is transferred to UFC1, it is ligated to target proteins *via* the UFM1-specific E3 ligase UFL1, which functions in complex with UFBP1 and CDK5RAP3 (202).

### ATG8/ATG12 E1

Among noncanonical E1s, autophagy-related protein 7 (ATG7) is unique in its ability to activate two Ubls, ATG8 and ATG12, which function in distinct cellular pathways (203). ATG7 is central to autophagy, facilitating intracellular turnover by catalyzing the conjugation of ATG8 to phosphatidylethanolamine on phagophore membranes (204, 205, 206). Once lipidated, ATG8 regulates autophagosome dynamics, modulating their size, interacting directly with cargo or autophagy receptors, and facilitating autophagosome-lysosome fusion for degradation (207, 208). In contrast, ATG12 does not undergo lipidation but instead functions in complex with ATG12 and ATG16L1, promoting LC3 lipidation, phagophore membrane elongation, and facilitating ATG8 conjugation through E3-like activity (209, 210, 211, 212). Additionally, ATG12 plays noncanonical roles in mitochondrial homeostasis and apoptotic regulation, influencing cell fate decisions (213, 214). Given its essential role in cellular homeostasis, ATG7 loss or dysregulation leads to severe consequences, including neurodegenerative diseases and cell death due to the accumulation of damaged organelles and cellular debris (215, 216, 217, 218).

#### A distinct architecture

ATG7 functions as a homodimeric protein, with each monomer consisting of ∼700 residues with two domains connected by a short linker (Fig. 5*C*) (219). The NTD (residues 1-288) is structurally distinct from both canonical and noncanonical E1s, lacking significant sequence homology. The CTD contains the AD (residues 294-572) and an extreme C-terminal domain (ECTD; residues 573-630). Like other noncanonical E1s, ATG7’s AD shares structural similarity with the prokaryotic MoeB and ThiF enzymes. The catalytic cysteine (Cys507) is located on the crossover loop (residues 473-511). When homodimerized, ATG7 adopts a shape resembling a bird in flight, with the NTDs forming the “wings” and the static AD comprising the “body” (PDBs: 3VH2) (219).

Binding of a Ubl to ATG7 induces significant conformational changes at the crossover loop, allowing the C terminus of the Ubl to pass under the loop toward the ATP-binding site (PDB: 3VH3). A series of stabilizing interactions form between residues of the AD and the C terminus of ATG8. Disrupting these interactions prevent adenylation but do not significantly impact binding affinity, suggesting a role in stabilizing the C-terminal tail of ATG8 or ATG12 for catalysis (219).

The ECTD of ATG7, which is partially unstructured in *apo* structures, becomes more ordered upon ATG8 binding. This region is critical for ATG8 recognition, as truncations of five and 13 C-terminal residues result in severe to complete loss of binding. A solution structure of ATG8 in complex with the last 30 residues of ATG7 (PDB: 2LI5) revealed a winding interaction that buries ∼1490 Å2 of surface area through a combination of hydrophobic and ionic contacts. Loss of these interactions abolishes ATG7-ATG8 binding, highlighting their critical role in ATG8 recruitment and activation. These findings support a model in which the ECTD of ATG7 facilitates ATG8 recruitment—captured in a proposed intermediary state in PDB: 5YEC—while contacts within the AD stabilize the Ubl C terminus for adenylation (220).

Following thioester bond formation, ATG12 and ATG8 must be transferred to their respective E2s, ATG10, and ATG3 (204, 206). Unlike canonical E1s that recruit E2s *via* a UFD, ATG7 utilizes its NTD for E2 recognition (219, 221). Specifically, structures of ATG7 in complex with ATG10 or ATG3 reveal an interface formed by the underside of the NTD, the side of the CTD, and the NTD-CTD junction (“underwing”), which interact with the backside β-sheet of each E2 (PDBs: 4GSL, 4GSK).

Similar to the *trans*-binding mechanism observed in UBA5, ATG7 transfers ATG12 and ATG8 to their respective E2s *via* a *trans* mechanism, where the Ubl thiolated by one CTD protomer is transferred to the E2 bound to the NTD of the opposite protomer (Fig. 6*C*) (219, 222, 223). However, the molecular basis for how ATG7 transfers its Ubls to their cognate E2s remains unclear. Future studies that capture the ATG3-ATG7∼ATG8 and ATG10-ATG7∼ATG12 complexes may elucidate the mechanism behind this specificity. Once transferred to their respective E2s, ATG10 catalyzes the conjugation of ATG12 to ATG5, forming a complex with ATG816L1 that acts as an E3-like ligase for ATG8-ATG3, facilitating ATG8 conjugation to phosphatidylethanolamine (203).

## Prokaryotic E1-like enzymes

The ADs of E1 enzymes and Ubls have bacterial origins, evident in MoaD and its activator MoeB, as well as the thiamine biosynthesis protein S and its activator ThiF. MoaD and ThiS share a Ubl fold (and the C-terminal Gly-Gly found in Ub) and function as sulfur carrying proteins for the synthesis of molybdopterin and thiazole, respectively. MoeB and ThiF are homodimeric proteins with structural and mechanistic similarities, sharing 46% sequence identity (53, 224). Both subunits of MoeB and ThiF can bind ATP-Mg^2+^ and their respective Ub-fold containing proteins. The structural and mechanistic features critical for adenylation are highly conserved, with the ADs of eukaryotic E1 enzymes closely superimposing with those of the bacterial MoeB and ThiF. This is especially evident in the noncanonical E1s, where both protomers are capable of catalyzing adenylation. Thus, MoeB and ThiF exemplify the core machinery for binding and adenylating Ubls (53).

### A newly characterized E1-E2–like fusion

Recent studies have expanded our understanding of the evolutionary trajectory of Ubl cascades, revealing an ancient bacteriophage defense system with structural parallels to an E1-E2 fusion protein (225). Bacteria must rapidly respond to phage infections, but these defense mechanisms require tight regulation to prevent premature cell death (226). One such regulatory protein, cGAS/DncV-like nucleoidyltransferases (CD-NTases)-associated protein 2 (Cap2), plays a key role in bacteriophage defense. Within cyclic oligonucleotide-based antiphage signaling systems, Cap2 regulates programmed cell death to abort phage proliferation, although the precise molecular mechanism underlying its function has only recently been uncovered.

Structurally, Cap2 shares a striking resemblance to an E2-bound ATG7 (Fig. 5*D*) (PDB: 7TO3) (225). Cap2 incorporates an N-terminal E2-like domain and a linking “wing” domain that mirrors the NTD of ATG7. The wing domain serves a similar role in stabilizing the N-terminal E2-like domain, akin to how the NTD of ATG7 secures ATG3 or ATG10 in their respective complexes (PDBs: 4GSL, 4GSK) (221). The N-terminal E2 domain folds into the “underwing” region created by the linking wing domain and AD.

### CD-NTase, the non-Ubl substrate

The substrate of Cap2 is CD-NTase, a relatively large protein (∼400 residues) that lacks the conserved β-grasp fold found in most Ubls (227). Cap2 facilitates CD-NTase binding through an interface between the helical face of its E2-like domain (from one protomer) and the AD of the other. Interactions within the AD domain guide the C-terminal tail of the CD-NTase into the active site, where adenylation and subsequent transfer to the E2-like domain drive its conjugation to an unknown target, ultimately promoting cGAMP synthesis (225). Cap3, a regulatory protein homologous to deubiquitinating enzymes, antagonize this process by cleaving CD-NTase from its conjugation target, likely serving as a negative regulator of cyclic oligonucleotide-based antiphage signaling systems (225, 228).

At the time of writing, Cap2 is the only known Ubl-transferase system that activates a non–β-grasp protein, suggesting that a broader spectrum of Ubl-like activation, conjugation, and ligation systems may exist but remain undiscovered due to their utilization of noncanonical substrates. Further studies are needed to identify these systems, as well as clarify the evolutionary relationship between these prokaryotic systems and eukaryotic Ubl cascades.

#### Therapeutic implications and small molecule targeting

Given their central role in cellular pathways, involvement in disease pathogenesis, and well-characterized enzymatic activity, E1 enzymes have emerged as promising therapeutic targets. Small-molecule inhibitors that mimic Ubl-adenylate intermediates have been developed both as research tools and potential anticancer agents.

One such compound, MLN-4924 (also known as pevonedistat), is a first-in-class E1 inhibitor shown to specifically inactivate NEDD8 E1 (159). This specificity has made MLN-4924 a useful tool in research for cullin functional studies, as well as an attractive compound for chemotherapies (229, 230). Several clinical trials utilizing pevonedistat have been completed and several are ongoing, but it has not yet been approved outside of these trials (ClinicalTrials.gov Identifiers: NCT00911066, NCT01862328, NCT03772925).

A related compound, TAK-243 (also known as MLN-7243), has been found to have nanomolar affinity for several E1s, including UBA1, NAE, UBA6, UBA7, and ATG7 (231, 232, 233). It is currently under clinical investigation for advanced cancers, refractory acute myeloid leukemia, and myelodysplastic syndromes (ClinicalTrials.gov Identifiers: NCT06223542, NCT03816319). However, a clinical trial evaluating TAK-243 for solid tumors was terminated, likely due to a combination of disease progression and adverse effects (NCT02045095).

Mechanistically, these compounds bind to the adenylation pocket, where it reacts with the catalytic cysteine, forming an adenylate mimetic that inhibits enzyme activity (PDBs: 3GZN, 5L6I). A related inhibitor, TAK-981, was developed to target the SUMO E1 SAE and is also undergoing clinical trials (NCT04381650, NCT04065555) (234). Other compounds have been developed that are specific for UBA1 (PYZD-4409) or UBA5 (ADS and a currently unnamed organometallic inhibitor), but these have not advanced to clinical trials (235, 236, 237).

Advancements in structural and mechanistic studies of E1 enzymes have paved the way for highly selective allosteric inhibitors, exemplified by COH000, an SAE inhibitor (238, 239). During the catalytic cycle of SAE, a cryptic binding pocket is transiently exposed, where COH000 covalently binds to a cysteine residue (Cys30 in hSAE2). This irreversible modification stabilizes an over-rotation of the SCCH domain, effectively locking SAE in a catalytically inactive state where it cannot form the SUMO thioester bond (PDB: 6CWY).

Given that allosteric inhibition has been successfully leveraged in other enzyme classes, drug screening efforts may identify additional E1 inhibitors that utilize a similar mechanism. Notably, this type of regulation is also observed in nature, as previously described with InsP6 inhibition of UBA6 (58). However, as of this writing, COH000 has not yet advanced into clinical trials.

#### Future directions

E1 enzymes are the keystones of Ubl cascades, orchestrating countless cellular processes across all domains of life. Significant progress has been made in understanding their molecular mechanisms, particularly how they maintain specificity for Ubls and E2s despite a high degree of conserved architecture. However, many aspects of E1 catalytic mechanisms remain unresolved, leaving room for new discoveries. The “resolution revolution” in cryo-EM provides an unprecedented opportunity to push the boundaries of E1 structural biology, potentially uncovering previously undetected interfaces and binding pockets that could be leveraged for rational drug design.

Beyond eukaryotic systems, investigations into the mechanism of E1-E2 fusion enzymes in prokaryotes—such as Cap2—demonstrates a broader, more ancient role for activation systems that utilize non-Ubl substrates. These findings expand our understanding of cellular regulation and may inspire novel antibiotic strategies targeting bacterial E1 pathways. Additionally, they raise the exciting possibility that eukaryotic E1 enzymes engage with unexpected, noncanonical substrates. Such a discovery could open new avenues for treating human diseases and underscores the need to deepen our understanding of canonical and noncanonical E1 cascades.

## Conflict of interest

The authors declare that they have no conflict of interest with the contents of this article.
